# Automated classification of mixed populations of *Aedes aegypti* and *Culex quinquefasciatus* mosquitoes under field conditions

**DOI:** 10.1186/s13071-024-06417-z

**Published:** 2024-09-19

**Authors:** Fábio Castelo Branco Fontes Paes Njaime, Renato Cesar Máspero, André de Souza Leandro, Rafael Maciel-de-Freitas

**Affiliations:** 1 Programa de Pós-graduação em Vigilância e Controle de Vetores, Instituto Oswaldo Cruz, Fiocruz - IOC, Rio de Janeiro, RJ Brazil; 2 Centro de Controle de Zoonoses da Secretaria Municipal de Saúde de Foz do Iguaçu, Paraná, Brazil; 3 Laboratório de Mosquitos Transmissores de Hematozoários, Instituto Oswaldo Cruz, Fiocruz-IOC, Rio de Janeiro, RJ Brasil; 4 Bernhard Nocht Institute for Tropical Medicine, Hamburg, Germany

**Keywords:** *Aedes aegypti*, *Culex quinquefasciatus*, Smart trap, Wing beat, Surveillance

## Abstract

**Background:**

The recent rise in the transmission of mosquito-borne diseases such as dengue virus (DENV), Zika (ZIKV), chikungunya (CHIKV), Oropouche (OROV), and West Nile (WNV) is a major concern for public health managers worldwide. Emerging technologies for automated remote mosquito classification can be supplemented to improve surveillance systems and provide valuable information regarding mosquito vector catches in real time.

**Methods:**

We coupled an optical sensor to the entrance of a standard mosquito suction trap (BG-Mosquitaire) to record 9151 insect flights in two Brazilian cities: Rio de Janeiro and Brasilia. The traps and sensors remained in the field for approximately 1 year. A total of 1383 mosquito flights were recorded from the target species: *Aedes aegypti* and *Culex quinquefasciatus*. Mosquito classification was based on previous models developed and trained using European populations of *Aedes albopictus* and *Culex pipiens*.

**Results:**

The VECTRACK sensor was able to discriminate the target mosquitoes (*Aedes* and *Culex* genera) from non-target insects with an accuracy of 99.8%. Considering only mosquito vectors, the classification between *Aedes* and *Culex* achieved an accuracy of 93.7%. The sex classification worked better for *Cx. quinquefasciatus* (accuracy: 95%; specificity: 95.3%) than for *Ae. aegypti* (accuracy: 92.1%; specificity: 88.4%).

**Conclusions:**

The data reported herein show high accuracy, sensitivity, specificity and precision of an automated optical sensor in classifying target mosquito species, genus and sex. Similar results were obtained in two different Brazilian cities, suggesting high reliability of our findings. Surprisingly, the model developed for European populations of *Ae. albopictus* worked well for Brazilian *Ae. aegypti* populations, and the model developed and trained for *Cx. pipiens* was able to classify Brazilian *Cx. quinquefasciatus* populations. Our findings suggest this optical sensor can be integrated into mosquito surveillance methods and generate accurate automatic real-time monitoring of medically relevant mosquito species.

**Graphical Abstract:**

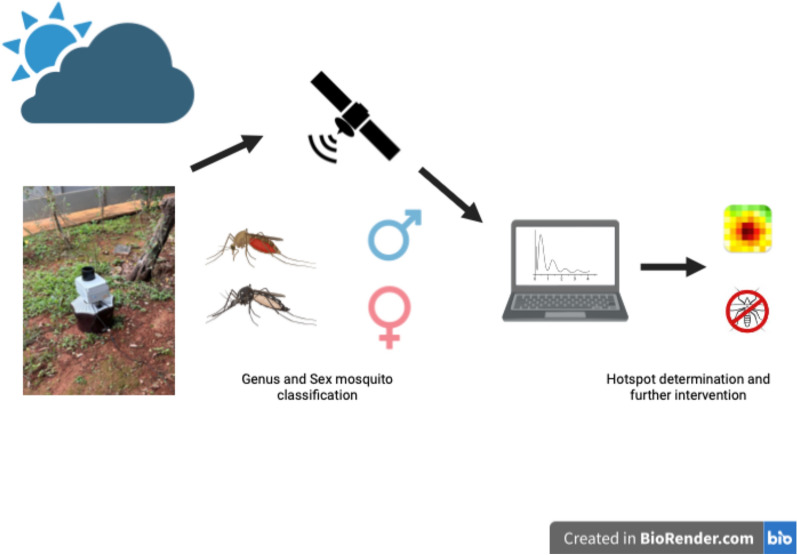

## Background

Emerging and re-emerging vector-borne diseases (VBD) represent significant global public health concerns, showing a rising incidence rate in endemic areas in the last decades [1, 2]. Equally important, VBD have expanded into new regions following an increase in the geographic distribution of their primary vectors [3]. Most of the VBD impacting public health systems are viruses and are transmitted by *Aedes* and *Culex* mosquitoes. Special interest is given to *Aedes aegypti*, *Ae. albopictus*, *Culex quinquefasciatus* and *Cx. pipiens*, species that are well adapted to living in association with humans. *Aedes aegypti* and *Cx. quinquefasciatus* are more abundant in urbanized areas and collected more frequently in the intradomestic environment of tropical regions. *Aedes albopictus* is more eclectic in biting behavior and habitat preferences, being found from suburban to peridomestic areas with high vegetation coverage [4–11].

Several biotic and abiotic factors can be linked to the recent expansion of VBD. Temperature seems to be the most limiting factor regarding vector distribution and thus disease transmission to temperate regions. Considering climate change and the expected increase in temperature in the next years, a subject for research is to foresee the impact of temperature rise on the transmission of arboviruses such as dengue (DENV), Zika (ZIKV), chikungunya (CHIKV), mayaro (MAYV), West Nile (WNV) and St. Louis encephalitis (SLE), to mention a few diseases notably transmitted by the three aforementioned mosquito species [12–17]. Climate change's impact is particularly significant in the twenty-first century, with some studies projecting a global temperature increase of 1.0–3.5 °C by 2100, intensifying arbovirus transmission but also expanding their occurrence to areas normally free from the viruses or with mild incidence [3]. Among the biotic factors influencing arbovirus transmission, human activities such as unplanned urbanization, construction of dams and irrigation schemes, increased global travel and trade, deforestation and the spread of insecticide resistance are factors playing a role in shaping mosquito and disease distribution [18–22].

We need to build and strengthen early warning systems and increase response capacities and preparedness for current and future threats. Regarding mosquito vectors, one of the greatest research gaps involves the implementation of a surveillance agenda that can efficiently determine seasonal mosquito population fluctuation and identify the high-risk areas in endemic metropolitan cities [23–25]. Undoubtedly, considering arbovirus epidemiology and disease dynamics, surveillance needs to determine the spatiotemporal risk in a timely manner, allowing further intervention (e.g. vector control activities such as ULV fogging, removal of potential breeding sites, community engagement, urban cleaning, etc.) to mitigate or halt transmission [26–28]. To estimate the sub-areas of a city with higher risk of disease transmission, vector sampling must be conducted citywide [26]. Regarding the biology of *Aedes* and *Culex* mosquitoes, performing mosquito sampling in the larval stage is unfeasible because of the many health agents required to sample every premise, the tedious nature of the work, which may impact the search effort, and a lack of long-term paramilitary organization to conduct larval surveys [23, 29]. Therefore, use of mosquito traps is recommended to make citywide sampling feasible. Additionally, it is well known that although immature stage monitoring might be simpler to set up, it lacks utility in estimating adult abundance and transmission risk due to poor correlation with egg, larval and pupal density indices [23, 25, 30]. A growing body of evidence shows that monitoring mosquito populations with adult traps better represents seasonal variations in mosquito abundance compared to indices derived from immature stage-based sampling like the House or Breteau Index [23, 30–33]. Myriad mosquito traps are available using different types of attractants and mechanisms, with their pros and cons [34]. Conventional traps with catch bags require periodic field collection and time-consuming entomological identification by an expert holding an appropriate taxonomic key, leading to delays in analyzing mosquito population dynamics [35]. Therefore, new approaches such as optical sensors combined with machine learning could provide almost real-time mosquito classification to support surveillance programs with timely determination of mosquito composition as soon as target species are trapped [36]. While historically wingbeat frequency has been the primary predictor variable, recent efforts have focused on improving classification methods to distinguish mosquito species, sex and even parity status [36]. Locally acquired field data on mosquito captures could be modeled with climatic records, traps, spatiotemporal localization and ecological features not only to improve remote mosquito classification in the field but also to develop real-time wireless entomological surveillance.

The development of automatic devices to further classify mosquito species is a growing field. It is important to test those devices under field conditions to provide a realistic challenge and reliable estimates. We report here an independent evaluation of a prototype optical sensor coupled to the BG-Sentinel [37, 38], a commercial mosquito trap commonly used in the field for trapping urban mosquito vectors. This evaluation included field sampling of *Aedes* and *Culex* mosquitoes in two sites in Brazil with subsequent adoption of previously established models to determine mosquito counts [36]. Herein, we report the effectiveness of the VECTRACK sensor in identifying and counting *Ae. aegypti* and *Cx. quinquefasciatus* in Rio de Janeiro and Brasilia using a classification model developed for *Ae. aegypti* and *Cx. pipiens* in Europe [36], highlighting the relevance of this model for genus classification.

## Methods

### Sensor and trap description

**Fig. 1:**
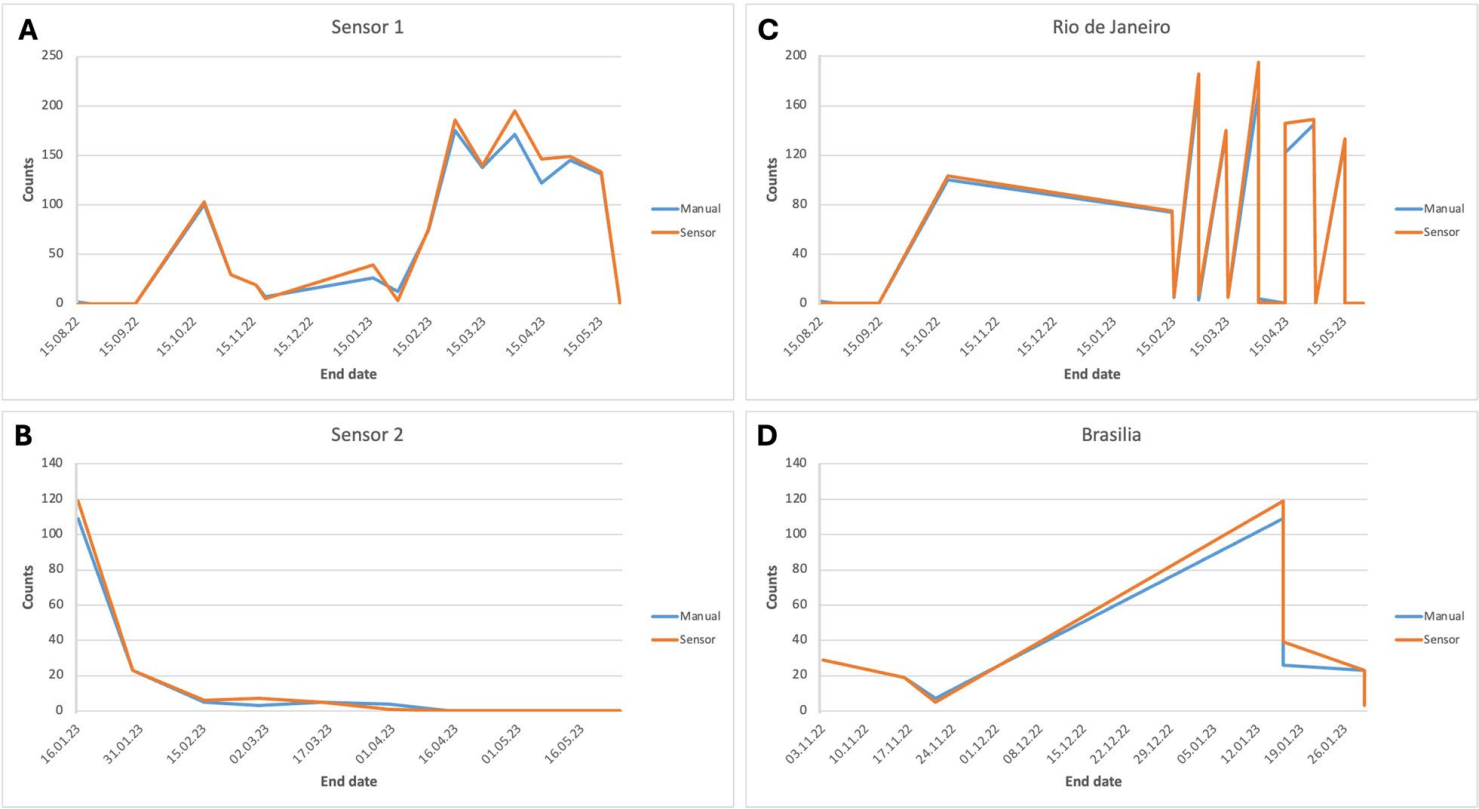
Time series plots representing the number of target mosquitoes (sensor count and manual count) per field assay. The x-axis indicates the end date of each field assay. **A** Sensor 1, **B** Sensor 2, **C** data gathered in Rio de Janeiro, **D** data gathered in Brasilia

The VECTRACK sensor, developed and manufactured by Irideon SL in Barcelona, Spain, was integrated with the entrance of a commercial BG-Mosquitaire suction trap from Biogents AG in Regensburg, Germany, as depicted in Fig. 1. Under a field setting, the BG-Mosquitaire trap captures mostly host-seeking mosquito females that are attracted to both visual and sensory cues, like its predecessor, BG-Sentinel [37, 38]. This trap is equally efficient in trapping both target species in field scenarios with high and low mosquito population density [37, 38]. The trap was equipped with a Biogents AG BGSweetscent chemical attractant sachet, and the fan-driven airflow within the trap was maintained at approximately 3 m/s in the downward direction. Mosquitoes flying in proximity to the sensor’s entrance funnel could be drawn in by the fan, detected by the sensor, and subsequently trapped in the catch bag within the trap body.

The sensor itself comprises an optical emitter panel and an optical receiver panel, positioned facing each other through a transparent flight tube with 105 mm diameter. The optical emitter consists of a two-dimensional (2D) array of 940-nm wavelength infrared light-emitting diodes (LEDs), while the optical receiver incorporates a 2D array of 940-nm photodiodes. The active length of the optical sensor in the downward direction is 70 mm. The output from the optical receiver undergoes amplification and is acquired by an analog-to-digital converter (ADC) with a sampling frequency of 9603 samples per second. Upon a mosquito entering the sensing volume, it triggers an automatic recording of up to 1024 samples, with a duration of up to 107 ms. Considering the typical duration of a mosquito’s flight is approximately 50 ms, this setup enables effective monitoring. Additionally, the sensor automatically timestamps each recording and captures the measured ambient temperature.

In this study, we targeted *Ae. aegypti* and *Cx. quinquefasciatus* from two field sites from Brazil, as described below. The classification of field-gathered specimens was based on previously developed and trained models. For *Ae. aegypti*, we used a model that was developed using both Spanish and Portuguese populations of *Ae. albopictus*, originally collected from Rubi, Barcelona (41°29′49″N; 02°02′05″E), and Algarve, Portugal (37°10′48″N; 08°22′22″E) [39]. The model used to classify Brazilian populations of *Cx. quinquefasciatus* was developed and trained for Spanish populations of *Cx. pipiens* obtained from Cerdanyola del Vallés, Barcelona (41°29′10″N; 02°04′29″E) [39].

### Data acquisition process

The two available VECTRACK sensors were exposed to a total of 28 non-simultaneous assays between July 2022 and May 2023. From those 28 assays, 21 were done in Rio de Janeiro and 7 in Brasilia. The two sensors were rotated in both cities to avoid any bias possibly related to specific geographic condition. Each assay consisted of exposing the VECTRACK sensor and BG-Mosquitaire to the field conditions reported above for a period ranging from 12 to 15 consecutive days.

### Study area and field trial conditions

The sensors were installed in two Brazilian cities: Rio de Janeiro and Brasilia. In Rio de Janeiro, the sensors were installed in a commercial company in Jacarepaguá (22°58′35″S; 43°24′59″W), a neighborhood characterized by vegetation coverage, mild mean temperature and presence of *Ae. albopictus* [7]. In Brasilia, the sensors were installed at the National Health Surveillance Department (15°48′53″S; 47°54′59″W), a highly urbanized area with almost no vegetation coverage. In each field site, the two sensors were installed indoors and outdoors to capture the diversity of mosquito vectors in the surroundings. Traps remained in the field for periods between 12 and 15 days, and all assays were conducted in an approximately 1 year period. The catch bags of the BG-Mosquitaire were inspected twice per week, i.e. around four times per assay. Traps remained plugged into power during the assays. The insects were identified using a Zeiss stereomicroscope and recorded considering the sensor and its location. The taxonomic identification of mosquito vectors was conducted using the appropriate local entomological keys [40]. A taxonomic identification of non-target species up to species level was out of the scope of this article.

### Data analysis of sensor classification in the field

Two main functions of the automated sensor were assessed: (i) the ability of the system to discriminate *Ae. aegypti* and *Cx. quinquefasciatus* target mosquitoes from non-target insects that were accidentally trapped and (ii) the ability of the system to correctly discriminate target mosquito species' genus and sex. The potential relationship between sensor count (mosquitoes counted by the sensor) and manual count (mosquitoes counted by manual inspection) was evaluated through correlation and linear regression analyses, with the results depicted using both time series and scatter plots for each collection cycle.

Pearson correlation coefficient (*r*) and associated *P*-values were calculated to assess the strength and significance of the relationship between the two variables. Additionally, regression coefficients, including the coefficient of determination (*R*^2^), linear slope and intercept, were computed to evaluate how well sensor count predictions aligned with manual counts. A regression slope greater or less than one indicated whether sensor counts tended to be higher or lower, respectively, than manual counts on average.

We used confusion matrix to evaluate the accuracy, sensitivity, specificity and precision of the classification tasks proposed. One confusion matrix was developed for each classification task, which involved classifying (i) target or non-target species, (ii) *Aedes* or *Culex*, (iii) *Ae. aegypti* sex and (iv) *Cx. quinquefasciatus* sex. For each confusion matrix, we determined the number of true positives (TP), true negatives (TN), false positives (FP) and false negatives (FN) using the manual classification as the reference gold standard. TP and TN are the numbers of positive and negative cases respectively that the system classified correctly. FP is the number of negative cases that the system incorrectly classified as positive, and FN is the number of positive cases that the system incorrectly classified as negative. To calculate TP, TN, FP and FN for a particular class, this class was defined as the positive class and the other class(es) were defined as the negatives. For the positive class, TP equals the minimum common value of the sensor and manual count. If the sensor count was greater than the manual count, then the difference was taken as FP; otherwise, FP equaled zero. If the sensor count was less than the manual count, then the difference was taken as FN. TN is calculated by subtracting FP from the manual counts for the negatives. Accuracy means how often the classifier is correct and was measured as (TP + TN)/total samples. Sensitivity, also known as true-positive rate or recall, indicates the proportion of positives that are correctly classified by the system and is represented as TP/(TP + FN). Specificity, also known as true-negative rate, indicates the proportion of negatives that are correctly classified by the system and is estimated TN/(TN + FP). Finally, the precision evaluates when the sensor predicts the sample as positive, how often it is correct. Precision is determined by TP/(TP + FP).

## Results

### Manual target species identification

A total of 1300 mosquitoes were sampled in all 28 assays, resulting in an average of 46.4 mosquitoes trapped per assay. From the total mosquitoes sampled, 627 (48.2%) were *Cx. quinquefasciatus* (545 females and 82 males) and 662 (50.9%) *Ae. aegypti* (436 females and 226 males). We still sampled 11 *Ae. albopictus* (10 females and 1 male), corresponding to 0.8% of the sampled specimens. Regarding the two field sites, 1215 (93.5%) mosquitoes were captured in Rio de Janeiro and 159 in Brasilia (12.2%). Focusing on the target species, in Rio de Janeiro, 43.7 and 55.4% were *Ae. aegypti* and *Cx. quinquefasciatus*, whereas in Brasília these species represented 82.4 and 17.6%, respectively.

### Manual non-target insect species identification

During all the 28 field assays, a total of 8012 non-target insects were sampled. From this sum, 6826 (85.2%) were sampled in Rio de Janeiro, whereas 1186 (14.8%) were collected in Brasilia. Among the non-target sampled insects, most insects were flies and other small dipterans, although we did not conduct the appropriate taxonomic identification of non-targeted specimens up to species level.

### Automated detection of target mosquito species in the field

**Fig. 2:**
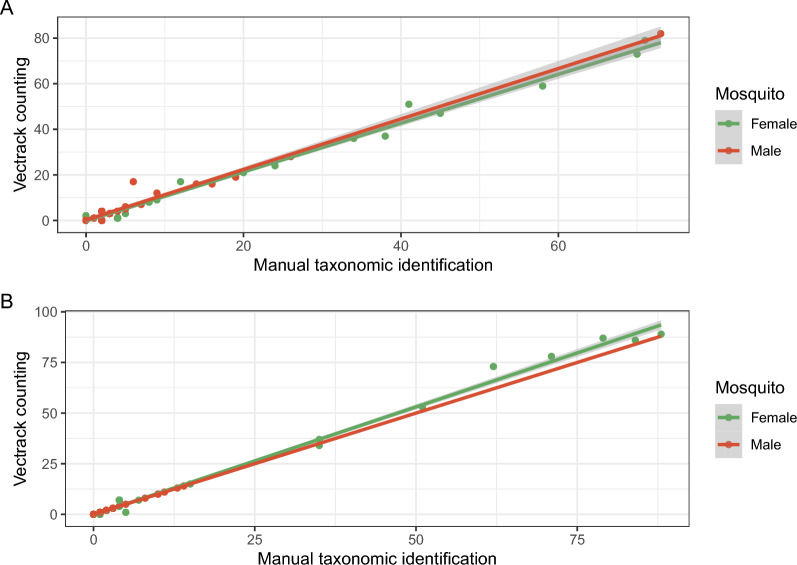
Scatter plot and linear regression of sensor count versus manual count for **A** *Aedes aegypti* and **B** *Culex quinquefasciatus* per mosquito gender showing the regression line equation (slope and y-intercept) and coefficient of determination. In **A**, the regression line for female and male is *y* = 1.0706x–0.1224 and *y* = 1.1085x + 0.1879, whereas the coefficient of determination is *R*^2^ = 0.992 and *R*^2^ = 0.9819 for female and male, respectively. In **B**, the regression line for female and male is *y* = 1.066x–0.248 and *y* = 0.9023x + 0.1432, whereas the coefficient of determination is *R*^2^ = 0.995 and *R*^2^ = 0.8897 for female and male, respectively

A remarkable overlapping between manual and sensor counts was observed in the two sensors used in the 28 field assays in both cities (Fig. 1). Linear regression analysis indicated a good fit of the linear regression line to manual count versus sensor count (Fig. 2). However, we should highlight the fit varied according to the species*genus combination. The coefficient of determination was > 0.99 in all cases, with the exception of males of *Cx. quinquefasciatus*, with a *R*^2^ equaling 0.889. Although we still consider it a satisfactory fit, further investigations should understand the hurdles in classifying *Cx. quinquefasciatus* males. Taking all the data together, i.e. using data from both sensors, both target species (*Ae. aegypti* and *Cx. quinquefasciatus*), plus males and females, a final coefficient of determination of 0.991 was observed, suggesting the VECTRACK sensor performed well in identifying mosquitoes.

### Confusion matrix and classification parameters

We developed one confusion matrix for each classification task. The most impressive results were obtained for classifying samples between target or non-target species, with accuracy, sensitivity, specificity and precision > 0.99 (Table 1). Those identified as target species were later classified under the genus *Aedes* or *Culex*. In this case, the VECTRACK sensor was able to accurately classify samples in 93.7% of the cases. In the genus classification, we observed the lowest sensitivity of the classification tasks we proposed: 92.1%. It indicates that around 8% of the samples were FN, i.e. the sensor classified them as *Culex* but they were *Aedes* after manual check. Regarding sex classification, better results were obtained for *Culex*, although similar results in sensitivity were observed for male and female sorting for both genera (Table 1).

**Table 1:** Summary of classification parameters based on confusion matrixes developed accordingly to the classification task

Classification task	Number of samples	Accuracy	Sensitivity	Specificity	Precision
Target and non-target species	9151	0.9987	1.000	0.9986	0.9915
Target species genus	1383	0.9372	0.9206	0.9471	0.9641
*Aedes aegypti* sex	731	0.9206	0.9408	0.8837	0.9368
*Culex quinquefasciatus* sex	660	0.95	0.9494	0.9534	0.9927

## Discussion

The development of automated identification sensors and systems designed for accurately identifying medically relevant mosquito species has seen significant advancement in recent years [41–43]. These ‘smart traps’ aim to provide reliable information regarding the identification of target species, ideally sorting them by sex. This is particularly important as it allows for the timely detection of female mosquitoes, a crucial parameter for estimating local disease transmission risk. In our study, we recorded the flight patterns of 10,534 insects in two distinct field sites in Brazil, characterized by different landscapes, abiotic conditions and arbovirus epidemiology. Among these, 1383 flight records belonged to medically important mosquitoes. To our knowledge, this report represents the first instance of utilizing sensors to classify target mosquito species and their sex within a dengue-endemic country.

Brazil has been experiencing over 1 million dengue cases annually since the late 2010s. By the end of April 2024, > 3 million dengue cases had been reported in the country, with concurrent circulation of CHIKV and ZIKV, albeit in smaller proportions [44, 45]. In this context, reliable tools for rapidly classifying field-caught mosquitoes are essential for public health management in endemic areas. Immediate identification of disease vectors can promote timely interventions. For instance, Brazil has been investing in strengthening its surveillance capacity for arboviruses. The city of Foz do Iguaçu has developed an innovative surveillance network based on One Health principles, focusing on five main areas: (i) integrating previously sectorized field teams into a unified One Health team capable of performing multiple tasks after receiving household allowances for indoor inspections; (ii) adopting digital solutions to replace archaic practices such as recording field information on printed spreadsheets; (iii) empowering health agents and providing them with continuous training alongside local scientists; (iv) mobilizing communities through meetings at schools, churches and community centers; (v) conducting active surveys to gain a better understanding of dengue epidemiology [27, 46]. A cornerstone of this surveillance system involves implementing adult mosquito sampling through extensive citywide trapping [26]. This enables local public health managers to predict periods of increased dengue transmission based on entomological indicators estimated through adult mosquito sampling [26, 28]. Further investigations have revealed that dengue transmission within the city is highly structured [47]. Analyzing a time series from 2017 to 2022, we observed certain areas with a higher likelihood of dengue transmission [48]. Therefore, incorporating VECTRACK sensor into an ongoing surveillance system, such as the one developed in Foz do Iguaçu, could significantly enhance its impact. In the routine entomological surveillance of Foz do Iguaçu, several hundreds of mosquitoes are sampled monthly. Assuming the mass trapping conducted by the city consists of collections in the urban environment, the mosquito diversity is low, i.e. only three species are sampled: *Ae. aegypti*, *Ae. albopictus* and *Cx. quinquefasciatus*. The VECTRACK sensors would be particularly useful in settings where large quantities of specimens are collected, where individual insect identification by local entomologists might be unfeasible. Installing these sensors in more vulnerable areas for dengue transmission would enable local public health managers not only to identify mosquito species and sex but also to integrate them into the early warning system algorithm [49]. This would provide alerts and prompt further interventions in areas with, for example, a high concentration of female *Ae. aegypti* mosquitoes and nearby human populations [13, 50–53].

Some reports have shown effective automatic classification of mosquito genera [54], sex [41] or both [36], but only a few have been able to provide point-of-capture classification. The VECTRACK sensor was used in two provinces of Barcelona, Spain, after training and developing a machine learning model using the flight of laboratory-reared *Ae. albopictus* and *Cx. pipiens*. A balanced accuracy of 95.5 and 88.8% in discriminating target mosquitoes and classifying genus/sex was observed, respectively [39]. In a recent report using semi-field conditions, the ability of an optoacoustic smart trap in classifying *Ae. aegypti, Cx. quinquefasciatus* and *Anopheles stephensi* with accuracies > 90% was shown [55]. We conducted field capturing of mixed native populations of both *Ae. aegypti* and *Cx. quinquefasciatus* in two dengue-endemic cities and observed 99.87% accuracies for classifying target species. Furthermore, the VECTRACK sensor discriminated *Aedes* and *Culex* with an accuracy of 93.72%. We also showed > 92% accuracy in discriminating mosquito sex in each of the target genera.

One remarkable outcome of our field survey in Brazil is that we used the models developed in Barcelona for *Ae. albopictus* and *Cx. pipiens* [36]. At least two conclusions can be reached regarding this result. First, the machine learning-based model for *Ae. albopictus* worked well in classifying *Ae. aegypti* mosquitoes from two Brazilian populations. The model developed and trained with Spanish and Portuguese *Ae. albopictus* populations worked surprisingly well for Brazilian populations of *Ae. aegypti*. The second remark regards classifying *Cx. quinquefasciatus* using the model developed and trained using Spanish populations of *Cx. pipiens* [36]. *Culex pipiens* complex comprises *Culex pipiens pipiens*, which exists in two forms, pipiens and molestus, along with *Culex pipiens pallens*, *Cx. quinquefasciatus*, *Cx. australicus* and *Cx. globocoxitus*. While some members of the complex have restricted geographic distributions, *Cx. pipiens pipiens* and *Cx. quinquefasciatus* are widely distributed across urban and suburban temperate and tropical regions worldwide [56]. Our findings suggest the original models developed and trained for European native mosquitoes of the *Aedes* and *Culex* genera could be extrapolated to other genetically related mosquito species from different countries. In that scenario, a further next step would involve developing specific models for *Ae. aegypti* and *Cx. quinquefasciatus* to test whether an expected increase in model accuracy in classifying target species is achieved.

The lowest correlation between manual counting and the VECTRACK sensor was observed for *Cx. quinquefasciatus* males. If the coefficient of determination between manual records and the VECTRACK sensor was > 0.98 for *Ae. aegypti* males and females, as well as for *Cx. quinquefasciatus* females, an *R*^2^ of 0.889 was estimated for *Cx. quinquefasciatus* males. One hypothesis to explain this could be related to mosquito size. It is well known in vector biology that the wing size is a proxy of mosquito size. Furthermore, the size of an adult mosquito is influenced by the quality of the breeding site in which mosquitoes have developed. In highly competitive low-resource breeding sites, mosquito size will likely be smaller than if mosquitoes are reared in a low-competitive and high-resource environment. Regarding *Cx. quinquefasciatus*, this species often uses large water bodies full of organic material for egg-laying and by corollary larval rearing [4, 57, 58]. Therefore, considering larval nutrients might be abundant in those breeding sites, the size of adult *Cx. quinquefasciatus* males that were trapped could have impacted our estimates.

The study had further limitations that should be noted. One is that we did not estimate mosquito size by measuring wing length, a common proxy adopted in medical entomology, and therefore could not test whether the size of *Cx. quinquefasciatus* males could negatively impact the coefficient of determination between manual counting and the VECTRACK sensor [59]. Additionally, our field sampling lasted almost 1 year, and due to abiotic factors related to seasonality, a period of 2 years of monitoring would be recommended. Nevertheless, herein we show the VECTRACK sensor is able to operate and provide robust and reasonably good classification accuracy results (93.72%) for the target *Aedes* and *Culex* mosquito species over the sampling conducted in two dengue-endemic cities of Brazil. We recommend additional tests should be done under more realistic field scenarios such as urban landscapes and areas with higher mosquito biodiversity. Furthermore, developing and training models for other mosquito vector species would be valuable as well.

## Conclusions

The use of emerging technologies to improve mosquito surveillance and vector control is on the rise lately, and their use can supplement the response to mosquito-borne diseases outbreaks. Our data show the effectiveness of an optical sensor in classifying target vs. non-target insect species with an accuracy of 99.9% and a specificity of 99.9%. Using field-gathered data, we could differentiate *Aedes* and *Culex* mosquitoes with an accuracy of 93.7% and precision of 96.4%. Finally, we also determined sex differentiation for the two genera of interest: *Aedes* and *Culex*. The determination of sex in *Cx. quinquefasciatus* mosquitoes achieved an accuracy of 95%, whereas separating male and female *Ae. aegypti* was done with a 92.1% success rate. Remarkably, similar results were obtained in two different Brazilian cities, Rio de Janeiro and Brasilia, suggesting high reliability of our findings. One important feature to highlight is potential extrapolation of the original developed and trained models. The model developed and trained for European colonies of *Ae. albopictus* and *Cx. pipiens* presented high accuracy for Brazilian populations of *Ae. aegypti* and *Cx. quinquefasciatus*, respectively. Our findings show this optical sensor can be coupled with conventional traps and later integrated into mosquito surveillance methods to generate accurate automatic real-time monitoring of medically relevant mosquito species.

## Data Availability

All data supporting the findings of the study are available within the article.

## Abbreviations

DENV: Dengue virus
ZIKV: Zika virus
CHIKV: Chikungunya virus
OROV: Oropouche virus
WNV: West Nile virus
BG: Biogents
VBD: Vector-borne diseases
MAYV: Mayaro virus
ULV: Ultra-low volume
LED: Light-emitting diodes
ADS: Analog to digital converter
*R*^2^: Coefficient of determination
TP: True positive
TN: True negative
FP: False positive
FN: False negative
